# Whole-transcriptome sequencing analysis to identify key non-coding RNAs in the pathogenesis of pulmonary tuberculosis

**DOI:** 10.5588/pha.26.0020

**Published:** 2026-08-24

**Authors:** M.-B. Liu, Y.-L. Guo, D.-J. Wu, Q.-Q. Ma, T. Jiang, M. Lin

**Affiliations:** 1 The central laboratory of Guizhou aerospace hospital, Zunyi, China;; 2 Department of Pharmacy Guizhou aerospace hospital, Zunyi, China;; 3 Department of Respiratory Guizhou aerospace hospital, Zunyi, China.

**Keywords:** tuberculosis, mRNA, differential expression, biomarkers

## Abstract

**BACKGROUND:**

Non-coding RNAs (ncRNAs) have been shown to play essential roles in various human diseases, including pulmonary tuberculosis (TB). This study aims to investigate differentially expressed ncRNAs, explore their potential biological functions, and determine whether they can serve as biomarkers and therapeutic targets in TB.

**METHODS:**

Whole-transcriptome RNA sequencing was performed on blood samples from TB patients and healthy controls (n = 3 per group). Differentially expressed mRNAs, long non-coding RNAs (lncRNAs), circular RNAs (circRNAs), and microRNAs (miRNAs) were identified, followed by Gene Ontology and Kyoto Encyclopedia of Genes and Genomes enrichment analyses. A competing endogenous RNA (ceRNA) network was constructed, and hub genes were identified via protein–protein interaction network analysis using Cytoscape.

**RESULTS:**

A total of 507 differentially expressed mRNAs, 107 differentially expressed lncRNAs, 253 differentially expressed circRNAs, and 38 differentially expressed miRNAs were identified. Functional enrichment revealed involvement in pathways related to cellular processes, mTOR signalling, and endocytosis. A circRNA–miRNA–mRNA ceRNA network was constructed, and hub mRNAs including RACK1, EIF3G, and RPS15 were identified.

**CONCLUSION:**

It is suggested that differentially expressed ncRNAs may play a role in the pathogenesis of TB. The circRNA (hsa_circ_0003340) and the hub mRNA (RACK1) are potential candidates for further exploration as biomarkers for TB infection. Moreover, the ceRNA network constructed in this study may provide new insights into the regulatory mechanisms of TB.

Pulmonary tuberculosis (TB) caused by *Mycobacterium tuberculosis (M.tb)* complex is a leading cause of morbidity and mortality.^1–4^ Despite the availability of effective drug treatments, TB remains a major public health issue for many countries, particularly due to the emergence of drug-resistant TB.^5–7^ Therefore, it is important to understand the molecular mechanism of TB development, which may offer a novel medical target for the treatment and prevention. Traditionally, non-coding RNAs (ncRNAs) were characterised by its lack of protein-coding capacity. Recent advances have established that ncRNAs are functionally active and regulate the expression of protein-coding genes through a variety of mechanisms.^8^ Emerging evidence indicates that targeting ncRNAs could represent a novel therapeutic strategy against TB.^9^ ncRNAs primarily consist of circular RNA (circRNA), long non-coding RNA (lncRNA), and microRNA (miRNA). CircRNA is an endogenous biomolecule characterised by a covalently closed loop structure, lacking 5′ caps and 3′ tails, and is expressed across various organisms. The lncRNAs are a regulatory class of ncRNAs with a wide range of activities such as transcriptional and post-transcriptional regulation. Studies show that lncRNA participate in diverse cellular processes, including epigenetic regulation, transcription, translation, and RNA metabolism.^10^ They exert regulatory effects in several ways: for instance, by interacting with DNA-binding proteins, recruiting epigenetic complexes involved in processes such as DNA methylation, or serving as precursors for small RNAs, particularly miRNAs.^11^ In contrast, miRNAs are short ncRNAs that post-transcriptionally regulate gene expression by directing target mRNA for degradation or translational repression. Recent studies have discovered that most circRNAs are known to function as miRNA sponges, sequestering them and thereby preventing the binding of miRNA to their target mRNA.^12^

This study aimed to identify differentially expressed ncRNAs in TB patients via whole-transcriptome sequencing. We subsequently constructed a competing endogenous RNA (ceRNA) network to elucidate the roles of ncRNAs in TB. Our findings are expected to provide novel insights for discovering and developing new targets for TB.

## METHODS

All participants were recruited from Guizhou Aerospace Hospital. The diagnosis of TB patients was established based on clinical manifestations, bacterial culture, and radiographic findings, all of whom presented no major complications. Healthy controls had no prior history of TB, had a normal chest X-ray, and were concurrently enrolled from the same period.

### RNA extraction and sequencing

The whole-transcriptome sequencing work was completed by Sangon Biotech (Shanghai) Co., Ltd. Total RNA was extracted from blood samples using the TRIzol extraction kit (Sangon Biotech, B511311). The purity of RNA was detected using a Qubit 2.0 fluorometer (Invitrogen, Q32866), and the concentration and integrity of RNA were detected using a Qubit 2.0 RNA detection kit (Life Technologies, Q32855). A sample is deemed suitable for RNA library prep and sequencing only if it adheres to these quality specifications: minimum concentration of 100 ng/μL, total mass ≥1 mg, and an OD260/280 ratio between 1.8 and 2.0.

The VAHTS™ Stranded RNA-seq Library Prep Kit for Illumina® (NEB, USA) was used for ncRNAs sequencing. Take 10 μl of total RNA from each sample as the starting volume to construct an RNA library. The rRNA was first removed from the samples using Ribo-off rRNA Depletion Kit (Vazyme, NR406-01), and Frag/Prime Buffer was added to the reaction system to fragment the RNA. The fragmented RNA was first reverse-transcribed into first-strand cDNA using random hexamer primers. Subsequently, the second cDNA strand was synthesised in a reaction mixture containing buffer, dNTPs, RNase H, and DNA Polymerase I. The resulting double-stranded cDNA was then purified using a Qubit DNA assay kit. The library preparation continued with a series of steps including end repair, ‘A’-tailing, and adapter ligation. Following size selection, the second strand of the cDNA was digested with UNG enzyme. The final library was generated and amplified via polymerase chain reaction (PCR), and its quality was assessed. Ultimately, sequencing was performed on the Illumina platform.

### Differentially expressed ncRNAs analysis

The DESeq method was applied to calculate the differentially expressed ncRNAs between TB and healthy individuals. The criteria for selecting differentially expressed ncRNAs between TB and healthy included adjusted *P* value (FDR) < 0.05 and |log_2_ fold change| ≥ 1. The outcomes of the differentially expressed ncRNAs analysis were visually represented in a volcano plot using the ‘ggplot2’ package. Differentially expressed ncRNAs were categorised into circRNAs, mRNAs, miRNAs, and lncRNAs, respectively.

### Functional annotation analysis

To understand the potential functions of differentially expressed circRNAs, mRNAs, miRNAs, and lncRNAs, Gene Ontology (GO) and Kyoto Encyclopedia of Genes and Genomes (KEGG) pathways were performed by using the cluster profiler (3.0.5) package of R software.

### Construction of circRNA–miRNA–mRNA ceRNA network

Based on the ceRNA hypothesis, we integrated expression data and regulatory interactions of lncRNAs, circRNAs, miRNAs, and mRNAs to construct a ceRNA network. Briefly, we first retrieved circRNA genomic positions using circBase and then predicted potential miRNA binding partners using the Cancer-Specific CircRNA Databse. Subsequently, these predictions were merged with the results from miRanda. The network was built by connecting lncRNA–miRNA, circRNA–miRNA, and miRNA–mRNA pairs that share common miRNA nodes. The resulting network was assembled and visualised using Cytoscape (version 3.9.1). To enhance interpretability, distinct RNA categories were represented by different node shapes, and differential expression (up- or down-regulation) was indicated by colour coding.

### Construction of the PPI network and screening of hub genes

The protein–protein interaction (PPI) network was constructed using the Search Tool for the Retrieval of Interacting Genes/Proteins (STRING) database (http://string-db.org) and visualised with Cytoscape software (version 3.8.2). Subsequently, the CytoHubba plugin in Cytoscape was employed to calculate degree values via its degree algorithm, enabling the identification of hub genes from the PPI network.

### Statistical analysis

All statistical analyses were performed using R software. Differentially expression analysis of ncRNAs was conducted using the DESeq2 package (version 1.26.0). Multiple testing correction was carried out using the Benjamini–Hochberg procedure to control the false discovery rate (FDR). An ncRNA was considered significantly differentially expressed if it met both of the following criteria: adjusted *P* value (FDR) < 0.05 and |log_2_ fold change| ≥ 1.

### Ethical statement

The studies involving humans were approved by the Ethics Committee of the Guizhou Aerospace Hospital (Ethics No. 2024KY-11). The studies were conducted in accordance with the local legislation and institutional requirements. The participants provided their written informed consent to participate in this study.

## RESULTS

### Transcriptome data quality control

First, to ensure the accuracy of the transcriptome analysis, we filtered the raw sequencing data. Quality control results demonstrated good consistency in the sequencing data quality. And the results showed that in circRNA and lncRNA, 86234508, 73098110, and 85982850 clean reads were generated in three TB patients; in miRNA, 8091797, 5934636, and 9457502 clean reads were generated in three TB patients, and in the three healthy control groups, 8247566, 9204892, and 12299479 clean reads were generated. The above data all indicate that the sequencing results can be further analysed. Detailed quality control results are listed in Table 1.

**TABLE 1.** Data filtering of circular RNA (circRNA), long non-coding RNA (lncRNA), and microRNA (miRNA).

Sample	Raw_reads	Clean_reads	Clean_rate (%)	Clean Q30 (%)	Clean GC (%)
circRNA and lncRNA					
C1	86036004	82650496	96.07	95.49	58.34
C2	87883198	84456940	96.10	95.45	58.08
C3	79352546	76037900	95.82	95.48	58.85
TB1	89213946	86234508	96.66	95.50	58.13
TB2	76058520	73098110	96.11	95.42	57.15
TB3	89864242	85982850	95.68	95.08	57.52
miRNA					
C1	8594579	8247566	95.96	96.60	40.16
C2	9675214	9204892	95.14	96.53	40.06
C3	12871878	12299479	95.55	96.57	40.31
TB1	8518824	8091797	94.99	96.55	39.69
TB2	6283297	5934636	94.45	95.76	40.13
TB3	10164490	9457502	93.04	96.44	39.95

### Differentially expressed ncRNAs

**FIGURE 1.**
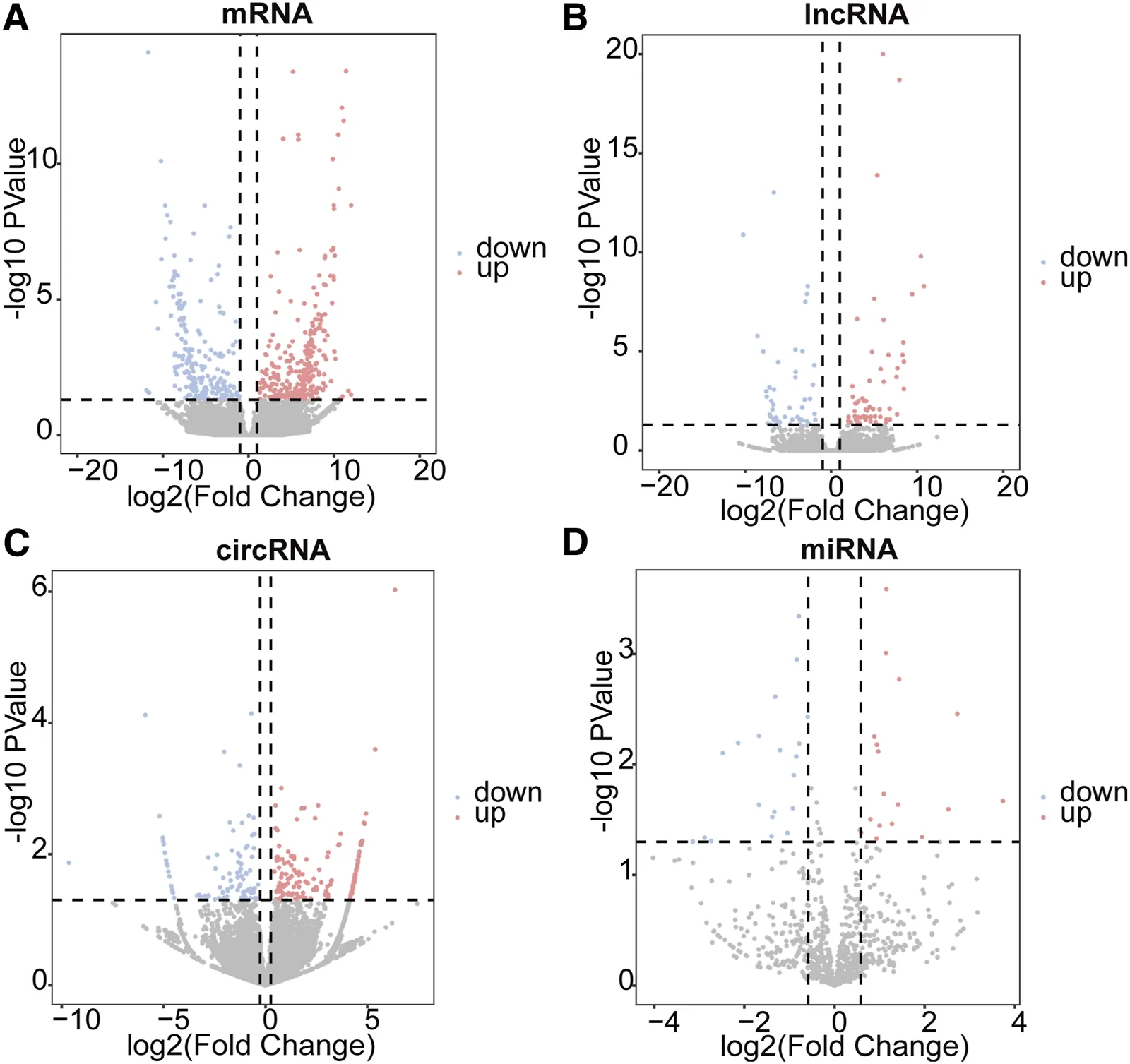
Four volcano plots. **A)** mRNAs, **B)** lncRNAs, **C)** circRNAs, and **D)** miRNAs showed differential expression. The X-axis represents log_2_ fold change, and the Y-axis represents negative log_10_ *P* value. Red dots indicate upregulated, blue indicate downregulated, and grey indicate not significant. Each plot displays data points distributed around a central region with varying significance levels. lncRNA = long non-coding RNA; circRNA = circular RNA; miRNA = microRNA.

To explore whether serum ncRNAs are potential biomarkers and therapeutic targets for TB infection (TBI), the landscape of expressed ncRNAs in serum was investigated by performing the VAHTS™ Stranded RNA-seq Library Prep Kit for Illumina® for six serum samples. As shown in Figure 1, differentially expressed ncRNAs were identified by comparing the TB group with the healthy control group, using the criteria of adjusted *P* value (FDR) < 0.05 and |log_2_ fold change| ≥ 1 to define statistically significant differential expression. In the TB group, 285 mRNAs were found to be upregulated and 222 downregulated; 59 lncRNAs were upregulated and 48 downregulated; 153 circRNAs were upregulated and 100 downregulated; and 18 miRNAs were upregulated while 20 were downregulated. Furthermore, the top five differential expressed ncRNAs were selected, and detailed information is presented in Table 2. And these diverse ncRNAs can serve as a reference for subsequent research.

**TABLE 2.** Top five differentially expressed mRNAs, circular RNAs (circRNAs), long non-coding RNAs (lncRNAs), and microRNAs (miRNAs).

Down	Log2 fold change	*P* value	Up	Log2 fold change	*P* value
mRNA					
SPOCK2	−27.92	1.62E−16	SECISBP2L	11.40	1.81E−18
ZEB2	−11.92	0.000198383	ATP2B4	11.68	0.000212736
ADD1	−11.71	1.82E−19	HEMGN	11.99	0.000321037
FYN	−11.68	0.000245973	NUP98	12.00	1.21E−12
IL6ST	−11.61	0.000260804	MAP2K3	27.91	3.90E−16
lncRNA					
GNAS	−26.32	1.81E−18	SBF2-AS1	8.782	2.51E−08
MSTRG.2493.3	−24.90	0.000212736	QARS	8.999	4.27E−12
ARHGAP4	−24.81	0.000321037	BIRC2	9.422	3.19E−14
GOLGA8B	−23.72	1.21E−12	MSTRG.5006.3	9.457	1.21E−12
AHNAK	−23.60	3.90E−16	ALMS1	9.531	1.04E−07
circRNA					
hsa_circ_0007715	−5.19	1.36E−02	hsa_circ_0084941	4.84	3.34E−03
hsa_circ_0006470	−4.99	7.59E−05	hsa_circ_0100093	4.86	3.45E−03
hsa_circ_0003340	−4.93	2.64E−03	hsa_circ_0092868	4.93	2.42E−03
hsa_circ_0095989	−4.88	5.63E−03	hsa_circ_0111224	5.38	2.54E−04
hsa_circ_0088398	−4.84	6.32E−03	hsa_circ_0011702	6.36	9.37E−07
miRNA					
hsa-miR-3684	−3.15	0.050	hsa-miR-145-3p	1.44	0.002
hsa-novel-65	−2.88	0.046	hsa-novel-127	1.94	0.045
hsa-miR-382-5p	−2.74	0.049	hsa-novel-17	2.53	0.025
hsa-miR-873-5p	−2.48	0.008	hsa-novel-122	2.72	0.003
hsa-miR-494-3p	−2.14	0.006	hsa-miR-548u	3.74	0.021

### GO and KEGG analysis of ncRNAs

**FIGURE 2.**
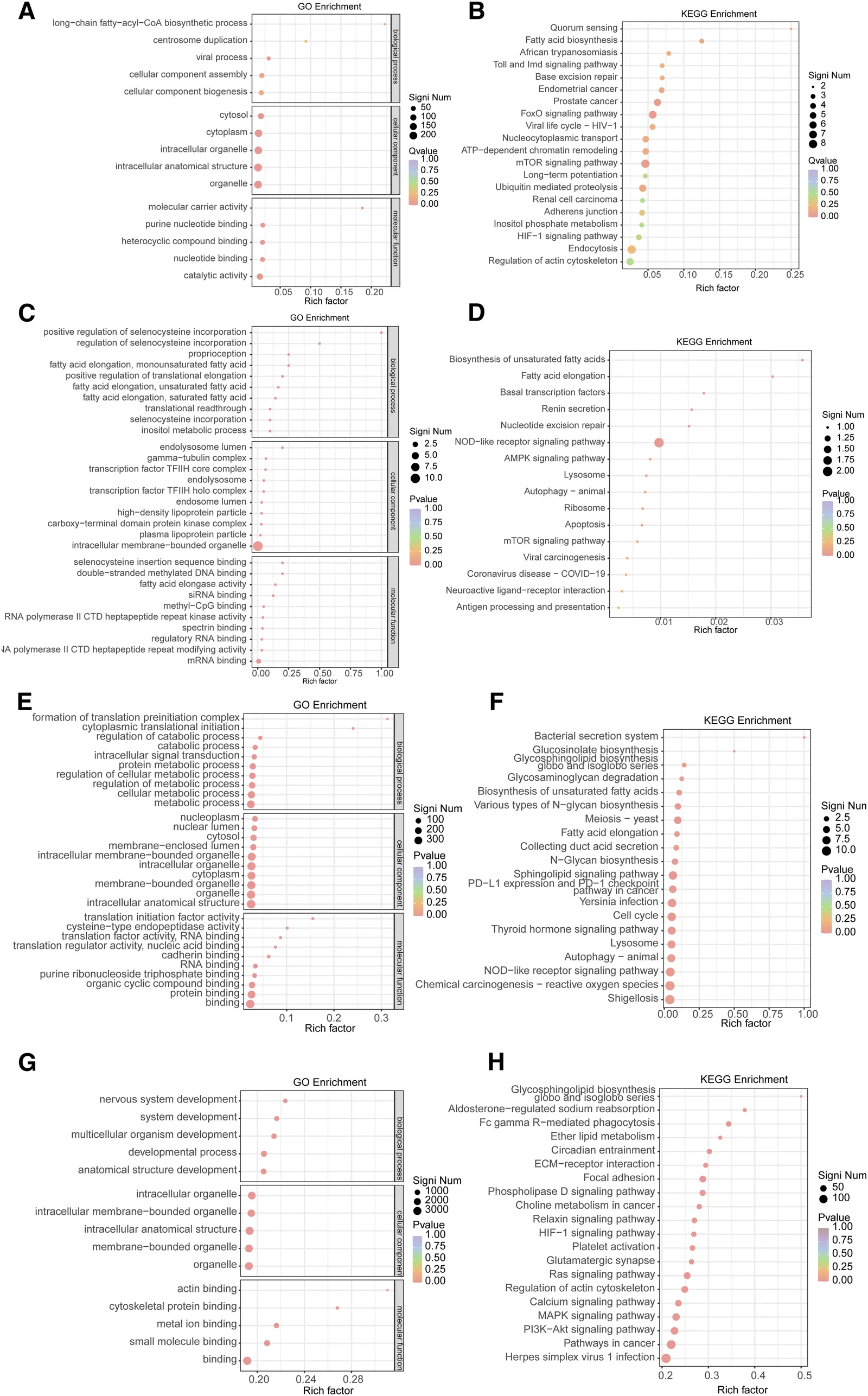
Four scatter plots show the enrichment results of differentially expressed circRNAs, lncRNAs, mRNAs, and miRNAs. **A)** Biological processes, cellular components, and molecular functions of circRNAs; **B)** KEGG enrichment of circRNAs. **C)** Biological processes, cellular components, and molecular functions of lncRNAs; **D)** KEGG enrichment of lncRNAs. Dots vary in colour and size, indicating different -log(FDR) values and sizes based on a legend. The x-axis shows rich ratio for all plots. **E)** Biological processes, cellular components, and molecular functions of mRNAs; **F)** KEGG enrichment of mRNAs. **G)** Biological processes, cellular components, and molecular functions of miRNAs; **H)** KEGG enrichment of miRNAs. Dots vary in colour and size, indicating different -log(FDR) values and sizes based on a legend. The x-axis shows rich ratio for all plots. circRNAs = circular RNAs; lncRNAs = long non-coding RNAs; miRNAs = microRNAs; KEGG = Kyoto Encyclopedia of Genes and Genomes; FDR = false discovery rate.

GO and KEGG can provide information from a biological function perspective, allowing us to understand how these differentially expressed ncRNAs function in vivo. GO enrichment analysis indicated that the biological processes associated with differentially expressed mRNA primarily included metabolic processes, cellular metabolic processes, and the regulation of metabolic processes. In terms of cellular components, they were mainly enriched in intracellular anatomical structures, organelles. Regarding molecular functions, the enriched terms included binding and organic cyclic compound binding (Figure 2A). KEGG pathway analysis further revealed that the differentially expressed mRNAs were significantly enriched in several pathways, such as Shigellosis and the nucleotide-binding oligomerisation domain (NOD)-like receptor signalling pathway (Figure 2B).

GO enrichment analysis indicated that the biological processes associated with differentially expressed miRNA primarily included in anatomical structure development, developmental process, and multicellular organism development. The cellular components included organelle and intracellular anatomical structure; and their molecular functions included binding, small molecule binding, etc. (Figure 2C). For miRNA, KEGG pathway analysis further include PI3K−Akt signalling pathway, MAPK signalling pathway, and Calcium signalling pathway (Figure 2D).

GO enrichment analysis indicated that the biological processes associated with differentially expressed circRNA included cellular component biogenesis and cellular component assembly; the cellular components involved included organelle and intracellular anatomical structure; and for the molecular functions, the main enrichment was in catalytic activity, nucleotide binding, etc. (Figure 2E). KEGG pathway included mTOR signalling pathway, FoxO signalling pathway, and Ubiquitin-mediated proteolysis (Figure 2F).

GO enrichment analysis indicated that the biological processes associated with differentially expressed lncRNA included inositol metabolic process, and translational readthrough; the cellular components included intracellular membrane-bounded organelle; and the molecular functions involved included mRNA binding, etc. (Figure 2G). KEGG pathways included NOD-like receptor signalling pathway in lncRNA (Figure 2H).

### Construction of a circRNA-mediated ceRNA network

**FIGURE 3.**
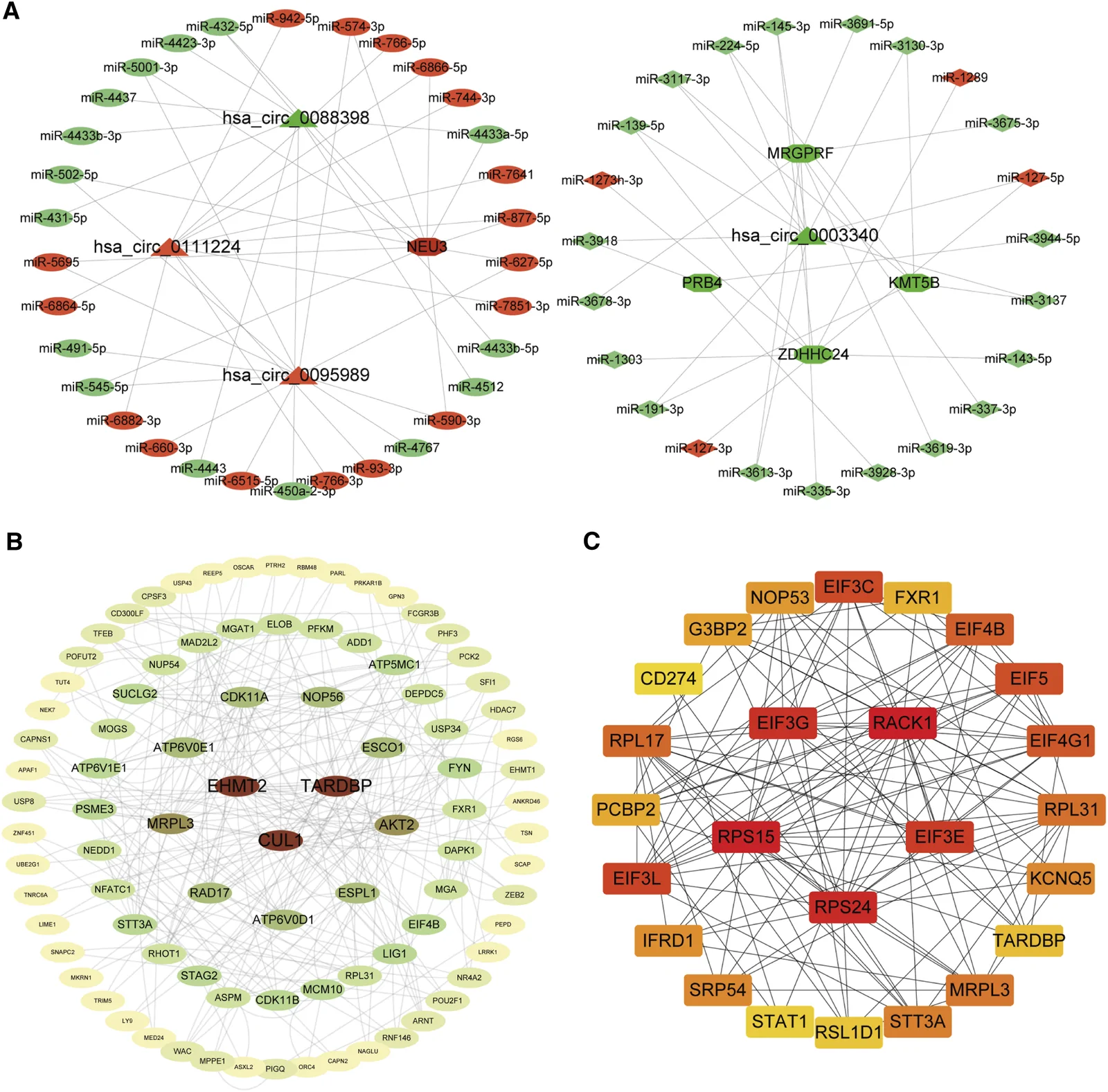
**A)** Network diagram showing relationships between circRNA, miRNA, and mRNA. Triangular nodes represent circRNA; diamond nodes represent miRNA; and circular nodes represent mRNA. Red indicates up-regulation, and green indicates down-regulation. Lines connecting nodes illustrate potential regulatory relationships. **B)** PPI network. The colour of the node changes from yellow to brown according to log_2_ (fold change). The frequency of each mRNA in the network determines the size of each node. **C)** Interaction of hub mRNAs. The top 25 hub mRNAs were chosen using the CytoHubba software’s degree algorithm. The degree score of each mRNA in the PPI network determines the size and colour of the nodes. circRNA = circular RNA; miRNA = microRNA; PPI = protein–protein interaction.

Research shows that the competitive ceRNA regulatory mechanism is involved in the occurrence and development of diseases. Thus, a circRNA-mediated ceRNA network was constructed to explore the potential regulatory roles of differentially expressed circRNAs in TB. The resulting network comprised 4 differentially expressed circRNAs (2 upregulated and 2 downregulated), 54 miRNAs, and 4 mRNAs, which together formed 48 circRNA–miRNA and 34 miRNA–mRNA interaction pairs (Figure 3A).

### Identification of hub mRNAs from the PPI network

To further investigate the functional interactions among differentially expressed mRNAs at the protein level, a PPI network was constructed using 108 differentially expressed mRNAs. The resulting network comprised 91 nodes and 472 edges, reflecting the complex regulatory relationships involved in biological processes such as signal transduction, cell cycle control (Figure 3B). To more clearly and intuitively identify genes of central importance, hub mRNAs were screened from the PPI network. The network was visualised using Cytoscape, and the CytoHubba plugin’s Degree method was applied to identify the top 25 hub mRNAs (Figure 3C). Among these, the most prominent genes (including RACK1, EIF3G, RPS15, RPS24, and EIF3E) are highlighted in the diagram, suggesting that they may play roles in the pathogenesis of TBI.

## DISCUSSION

*M.tb* can remain dormant in the body for extended periods. Early diagnosis and effective treatment are essential to prevent both further transmission and the emergence of drug-resistant strains. Advances in molecular biotechnology have revealed the significant involvement of ncRNAs in various disease pathways.^13^ Specifically, dysregulated expression of lncRNAs or circRNAs has been linked to the onset and progression of TB. However, their precise mechanisms and reliable biomarkers require further elucidation. This study aims to investigate differentially expressed ncRNAs, explore their potential biological functions, and determine whether they can serve as potential biomarkers in TB.

A total of 285 upregulated and 222 downregulated mRNAs, 59 upregulated and 48 downregulated lncRNAs, 153 upregulated and 100 downregulated circRNAs, as well as 18 upregulated and 20 downregulated miRNAs were identified in TB patients compared to healthy controls in our study. These differentially expressed ncRNAs could be considered as potential candidates for further exploration as biomarkers for TBI. GO and KEGG enrichment analyses were performed to explore the potential biological functions and signalling pathways associated with these differentially expressed ncRNAs. The results revealed distinct KEGG pathway enrichment patterns across different RNA types, providing biological insights into their potential roles in TB pathogenesis. Specifically, for mRNAs, the highly enriched pathways included NOD-like receptor signalling pathway. For miRNAs, significant enrichment was observed in pathways such as PI3K-Akt signalling pathway, MAPK signalling pathway, and Calcium signalling pathway. For circRNAs, the KEGG analysis showed high enrichment in the mTOR signalling pathway, FoxO signalling pathway, Endocytosis, Regulation of actin cytoskeleton, and Ubiquitin-mediated proteolysis. The innate immune system is the first host defence against *M.tb*. The recognition of this pathogen is mediated by several classes of pattern recognition receptors expressed on the host innate immune cells, including NOD-like receptor signalling pathway, PI3K-Akt signalling pathway, MAPK signalling pathway and mTOR signalling pathway.^14^

Previous studies have demonstrated that circRNAs can act as molecular sponges for miRNAs, thereby playing a crucial regulatory role in various physiological processes and infectious diseases, including TB, with miRNAs serving as essential bridges that connect different classes of RNAs.^15,16^ The circRNA–miRNA regulatory network has emerged as a widely recognised mechanism of gene expression control. Based on this rationale, a circRNA–miRNA interaction network was constructed using dysregulated circRNAs and TB-related miRNAs. Based on ceRNA theory, we constructed a regulatory network and identified several key target genes. Ultimately, a ceRNA network was constructed based on 4 circRNAs (hsa_circ_0111224, hsa_circ_0095989, hsa_circ_0088398, and hsa_circ_0003340), 54 miRNAs, and 4 mRNAs (NEU3, MRGPRF, KMT5B, and ZDHHC24), resulting in 48 pairs of circRNA–miRNA and 34 pairs of miRNA–mRNA relationships.

In this ceRNA network, Liang et al.^17^ showed that hsa_circ_0003340 sponged miR-615-5p to release PDX1, thereby elevating glutamine metabolism and facilitating tumour growth in oesophageal squamous cell carcinoma. Shen et al.^18^ demonstrated that MrgprF-forced expression inhibits tumour cell proliferation, migration, xenograft tumour growth, and metastasis. Hulen et al.^19^ found that KMT5B results in a skeletal muscle developmental deficit causing reduced muscle mass and body weight. To more clearly and intuitively identify genes of central importance, hub mRNAs (RACK1, EIF3G, RPS15, RPS24, and EIF3E) were identified, suggesting that they may play key roles in the pathogenesis of TB infection. Qu et al.^20^ reported that RACK1 functions as an endogenous host sensor for pathogens, and that pyroptosis induced by the interaction between EST12 and RACK1 plays a critical role in immunity against *M.tb*. Their study further revealed that EST12 triggers pyroptosis in macrophages through its interaction with RACK1. In addition, RACK1 has also been identified as a host factor involved in promoting viral replication through its interaction with viruses. This is consistent with the results we obtained. Hsa_circ_0003340 and RACK1 may potentially be further studied as biomarkers for TBI.

This study has several limitations. First, the included sample size is limited, which may affect the generalisability of the research findings. Additionally, this work represents only a preliminary exploration, and the transcriptomic sequencing results require further validation through in vivo or in vitro experiments. Finally, researchers can design primers and probes to collect patient blood samples for qRT-PCR verification, while also expanding the sample size to include different stages of TBI, various phases of medication, and cases with certain clinical complications, thereby further exploring these key ncRNAs as biomarkers and therapeutic targets.

## CONCLUSION

We identified differentially expressed ncRNAs in peripheral blood samples from TB and constructed a circRNA–miRNA network. Bioinformatics analysis suggested that hsa_circ_0003340 and RACK1 are potential candidates for further exploration as biomarkers for TBI.
